# Dissecting Disease-Suppressive Rhizosphere Microbiomes by Functional Amplicon Sequencing and 10× Metagenomics

**DOI:** 10.1128/mSystems.01116-20

**Published:** 2021-06-08

**Authors:** Vittorio Tracanna, Adam Ossowicki, Marloes L. C. Petrus, Sam Overduin, Barbara R. Terlouw, George Lund, Serina L. Robinson, Sven Warris, Elio G. W. M. Schijlen, Gilles P. van Wezel, Jos M. Raaijmakers, Paolina Garbeva, Marnix H. Medema

**Affiliations:** a Bioinformatics Group, Wageningen University and Research, Wageningen, The Netherlands; b Microbial Ecology, Netherlands Institute of Ecology (NIOO-KNAW), Wageningen, The Netherlands; c Microbial Biotechnology, Leiden Institute of Biology, Leiden, The Netherlands; d Biointeractions and Crop Protection, Rothamsted Research, Harpenden, United Kingdom; e BioTechnology Institute, University of Minnesota—Twin Cities, Falcon Heights, Minnesota, USA; f Bioscience, Wageningen University and Research, Wageningen, The Netherlands; University of California, Santa Cruz

**Keywords:** suppressive soils, functional amplicon, dom2BGC, 10× metagenomics, pathogenic fungi, *Fusarium*, wheat, biosynthetic gene cluster, disease suppression, nonribosomal peptide synthetase, rhizosphere, siderophores, software

## Abstract

Disease-suppressive soils protect plants against soilborne fungal pathogens that would otherwise cause root infections. Soil suppressiveness is, in most cases, mediated by the antagonistic activity of the microbial community associated with the plant roots. Considering the enormous taxonomic and functional diversity of the root-associated microbiome, identification of the microbial genera and mechanisms underlying this phenotype is challenging. One approach to unravel the underlying mechanisms is to identify metabolic pathways enriched in the disease-suppressive microbial community, in particular, pathways that harbor natural products with antifungal properties. An important class of these natural products includes peptides produced by nonribosomal peptide synthetases (NRPSs). Here, we applied functional amplicon sequencing of NRPS-associated adenylation domains (A domains) to a collection of eight soils that are suppressive or nonsuppressive (i.e., conducive) to Fusarium culmorum, a fungal root pathogen of wheat. To identify functional elements in the root-associated bacterial community, we developed an open-source pipeline, referred to as dom2BGC, for amplicon annotation and putative gene cluster reconstruction through analyzing A domain co-occurrence across samples. We applied this pipeline to rhizosphere communities from four disease-suppressive and four conducive soils and found significant similarities in NRPS repertoires between suppressive soils. Specifically, several siderophore biosynthetic gene clusters were consistently associated with suppressive soils, hinting at competition for iron as a potential mechanism of suppression. Finally, to validate dom2BGC and to allow more unbiased functional metagenomics, we performed 10× metagenomic sequencing of one suppressive soil, leading to the identification of multiple gene clusters potentially associated with the disease-suppressive phenotype.

**IMPORTANCE** Soil-borne plant-pathogenic fungi continue to be a major threat to agriculture and horticulture. The genus Fusarium in particular is one of the most devastating groups of soilborne fungal pathogens for a wide range of crops. Our approach to develop novel sustainable strategies to control this fungal root pathogen is to explore and exploit an effective, yet poorly understood naturally occurring protection, i.e., disease-suppressive soils. After screening 28 agricultural soils, we recently identified four soils that were suppressive to root disease of wheat caused by Fusarium culmorum. We also confirmed, via sterilization and transplantation, that the microbiomes of these soils play a significant role in the suppressive phenotype. By adopting nonribosomal peptide synthetase (NRPS) functional amplicon screening of suppressive and conducive soils, we here show how computationally driven comparative analysis of combined functional amplicon and metagenomic data can unravel putative mechanisms underlying microbiome-associated plant phenotypes.

## INTRODUCTION

Cereals are a staple food for the human population, with wheat as the most widely consumed cereal crop worldwide. It is estimated that up to 40% of crop yields are lost due to weeds, pests, and diseases (1). Pathogenic fungi are one of the major threats to agriculture. The genus Fusarium in particular is one of the most devastating groups of pathogens for a wide range of crops, including wheat (2, 3). Fusarium culmorum causes root rot and head blights in wheat and barley. It can kill plants at early stages of development or reduce their fitness and contaminate the grain with an arsenal of mycotoxins. Intriguingly, in some agricultural soils, root rot caused by *F. culmorum* does not occur or only to a small extent (4). This so-called soil disease suppressiveness is a phenomenon where plants show strongly reduced disease symptoms despite the presence of a virulent pathogen and conditions favorable for disease development (5). It is now well established that the soil and root microbiomes are essential for disease suppressiveness. In recent work, we performed an extensive screening of 28 soils for their suppressiveness to *F. culmorum* (4). We identified and confirmed, via sterilization and transplantation, that in four tested soils, the microbiome is associated with suppressiveness to *F. culmorum*. Subsequent comparative taxonomic analysis of the root-associated bacterial communities, aimed to identify differences in abundance or absence/presence patterns of specific genera, revealed only limited commonalities between the four suppressive soils. The overall aim of this study was to adopt a functional approach to generate hypotheses regarding putative mechanisms associated with the disease-suppressive phenotype.

Many microbe-microbe interactions are mediated by specialized metabolites with diverse functions, including inhibition of fungal growth (6). The production of these bioactive compounds is often encoded by biosynthetic gene clusters (BGCs): groups of physically clustered genes that encode molecular machineries such as nonribosomal peptide synthetases (NRPSs) and polyketide synthases (PKSs), which enzymatically assemble complex metabolites. Importantly, these BGCs are often discontinuously distributed across taxa due to high rates of horizontal gene transfer (7). Additionally, there may be functional redundancy due to overlapping biological activities between the products of different BGCs. Therefore, looking at BGC distribution patterns may help explain microbiome-associated phenotypes for which no clear taxonomic associations are identified. PKS and NRPS enzymes are often organized in multidomain modules, which each contain a set of enzymatic domains that extend the growing peptide or polyketide chain with a specific monomer during enzymatic assembly. Functional amplicon sequencing can target such domains using oligoprimers to amplify DNA from BGCs. Because the sequencing is highly selective, even BGCs from lowly abundant microorganisms can be detected by this technology (8, 9).

Here, we used NRPS amplicon screening for comparative functional analyses of a set of four suppressive and four conducive agricultural soils in the presence and absence of the pathogen *F. culmorum*. To facilitate this analysis, we introduce dom2BGC (code available at https://git.wur.nl/traca001/dom2bgc), a pipeline for extensive annotation of BGC-related amplicons. The amplicons are annotated based on similarity to domains in MIBiG and antiSMASH-DB, two large natural product BGC databases. For NRPS adenylation (A) domains, substrate specificities are predicted based on a newly built random forest classifier trained on the amplified region of these domains. When multiple samples are available, dom2BGC creates a co-occurrence network to aid in the detection of groups of amplicons that jointly originate from known or related BGCs. We applied dom2BGC and validated the annotation and clustering results with the high-quality metagenome of a selected sample enhanced using 10×-based read clouds. The results show siderophore BGCs as key candidates associated with disease suppressiveness of the soils against *F. culmorum*. The linked read metagenomic data set further revealed several additional BGCs that, based on their predicted functions, may be involved in the disease-suppressive phenotype. This study exemplifies how computationally driven analysis of combined functional amplicon and metagenomic data can unravel new candidate BGCs for further investigation and help to develop new hypotheses regarding the mechanisms underlying important microbiome-associated phenotypes.

## RESULTS AND DISCUSSION

### Identification of disease-suppressive agricultural soils.

**FIG 1:**
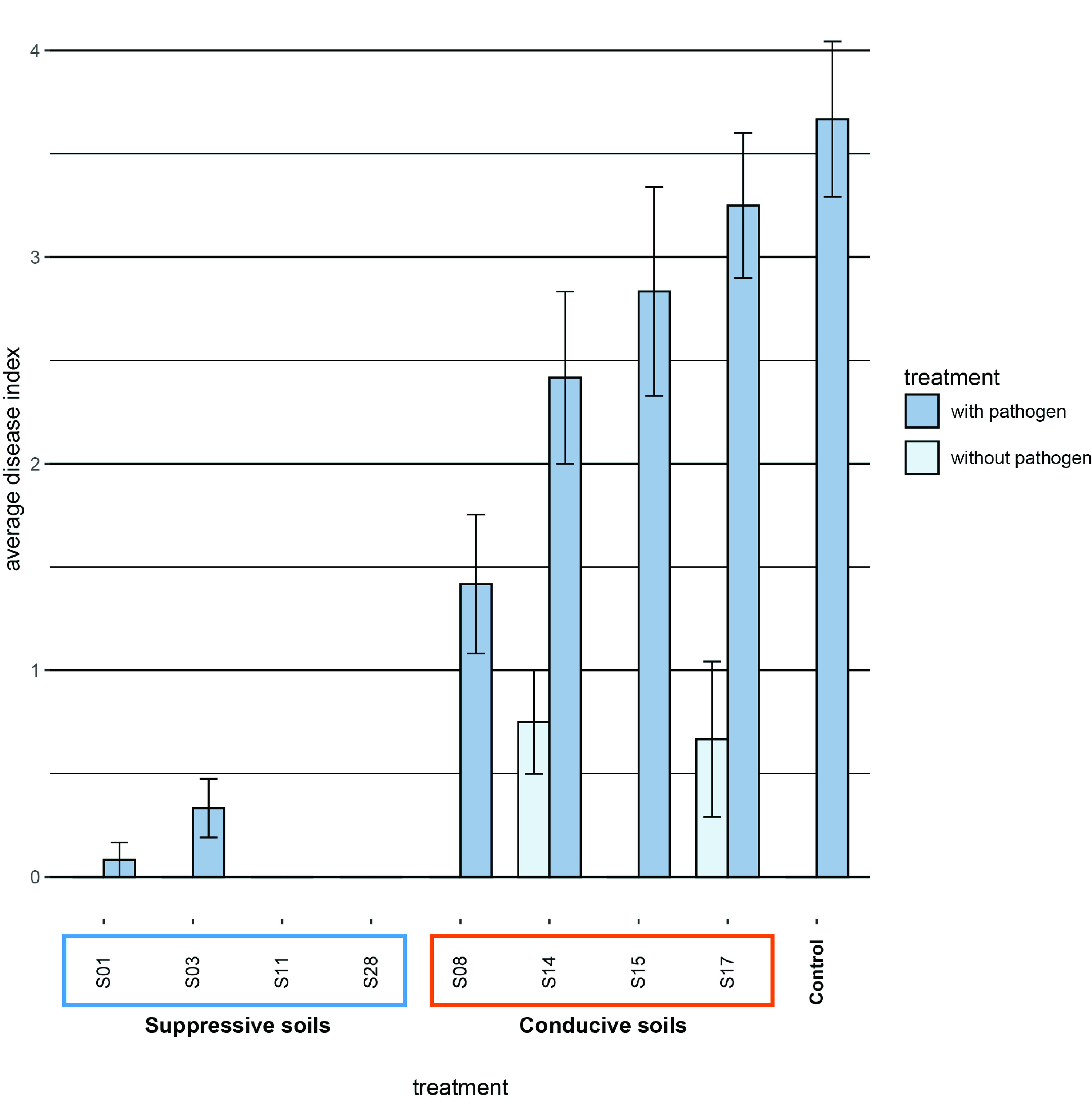
Disease index of Fusarium root rot disease of wheat grown in eight different agricultural soils. Four soils (S01, S03, S11, and S28) were classified as disease suppressive, and four soils (S08, S14, S15, and S17) were classified as disease conducive. Dark blue, inoculated with *F. culmorum*; light blue, noninoculated sterile BS dune soil was used as a control. The bars indicate the average disease indices, with the error bars representing the standard errors of the means (*n* = 12).

In our previous study (4), we tested 28 diverse field soils from the Netherlands and Germany for disease suppressiveness against Fusarium culmorum root rot of wheat. Based on these results, we selected four disease-suppressive (S01, S03, S11, and S28) and four disease-conducive (S08, S14, S15, and S17) soils for further analysis. For the amplicon-based analyses of the rhizosphere microbiome, we again performed disease suppressiveness assays on these eight soils. We observed no disease symptoms in two inoculated suppressive soils (S11 and S28) and only low levels of disease in the other two inoculated suppressive soils (S01 and S03). This clearly contrasts with the four conducive soils, where disease levels varied from moderate (S08) to high (S14, S15, and S17) (Fig. 1). In two of the conducive soils (S14 and S17), we also identified some mild disease symptoms in treatments without addition of the pathogen, indicating the presence of indigenous populations of *F. culmorum* or of other pathogens causing similar disease symptoms (Fig. 1, light blue bars). Altogether, these results confirm and extend the results of our previous study and show a clear distinction in phenotypes between the four suppressive and the four conducive soils.

### Functional amplicon sequencing uncovers novel NRPS domains from low-abundant bacteria in rhizosphere microbial communities.

As our previous 16S rRNA-based analysis of taxonomic similarities and differences between and across conducive and suppressive soils revealed that no taxa were unequivocally linked to disease suppression (4), we turned to functional amplicon sequencing to assess whether this could point to metabolites or classes of metabolites associated with the suppressive phenotype. The selective amplification of functional domains allows the capture of biosynthetic diversity found within a complex soil sample. Specifically, we used PCR amplification of A domains of NRPSs, which are involved in the production of several types of bioactive molecules that were previously linked to disease suppression, such as lipopeptides and siderophores. In NRPSs, the role of A domains is to recognize and activate amino acid substrates that are incorporated into the growing peptide (10). Based on their sequence, it is possible to predict their amino acid specificity and match them to databases of known or predicted BGCs.

Functional amplicon sequencing of adenylation domains across the four suppressive and four conducive soils produced 4,181,437 raw reads across all samples, which were used to identify association patterns of A domains across suppressive and conducive soils. One replicate from suppressive soil S28 (FC.1) (see Fig. S1 in the supplemental material) was removed from further analysis, because it produced significantly fewer reads than the other samples (12,380 reads, while the rest of the samples averaged 61,132 reads). Processing of the reads resulted in 3,396,393 reads mapping to 51,912 unique domains. Rarefaction analysis revealed that for most samples, diversity was sufficiently covered at ∼30,000 reads per sample (see Table S1).

10.1128/mSystems.01116-20.2 TABLE S1 Read counts per sample table pre- and postfiltering. Download Table S1, XLSX file, 0.1 MB. Copyright © 2021 Tracanna et al. 2021 Tracanna et al. https://creativecommons.org/licenses/by/4.0/ This content is distributed under the terms of the Creative Commons Attribution 4.0 International license.

10.1128/mSystems.01116-20.7 FIG S1 Rarefaction analysis of rhizosphere A domain amplicons. Sample rarefaction analysis showing number of unique AMP-binding domains (ordinate) at different rarefaction points (abscissa). One sample was clearly an outlier (the short pink line on the bottom) and was removed from the analysis. Download FIG S1, PDF file, 0.1 MB. Copyright © 2021 Tracanna et al. 2021 Tracanna et al. https://creativecommons.org/licenses/by/4.0/ This content is distributed under the terms of the Creative Commons Attribution 4.0 International license.

To facilitate linking amplicon sequences to specific BGCs, we generated a high-quality shotgun metagenome assembly of one sample from the rhizosphere microbiome of plants grown in soil S11. This soil was chosen because of its strong disease suppression in this study as well as in our previous experiments (4). To increase assembly contiguity, we made use of 10× linked read sequencing technology, which is able to generate many more contiguous contigs than what is possible with conventional metagenomics with comparable coverage. We used the dedicated cloudSPAdes 10× linked reads assembler on these data, which resulted in an assembly size of 2.2 Gb and an *N*_50_ of 2.8 kb for contigs >1 kb, with the largest contig measuring 1.3 Mb. Compared to the metaSPAdes equivalent assembly, which does not make use of the linked read information, we observed a considerable improvement in the *N*_50_ and assembly size for contigs >5 kb (7.3 kb for regular metaSPAdes assembly and 20.2 kb for cloudSPAdes), which makes the cloudSPAdes assembly more suited to obtain complete NRPS BGCs (11).

**FIG 2:**
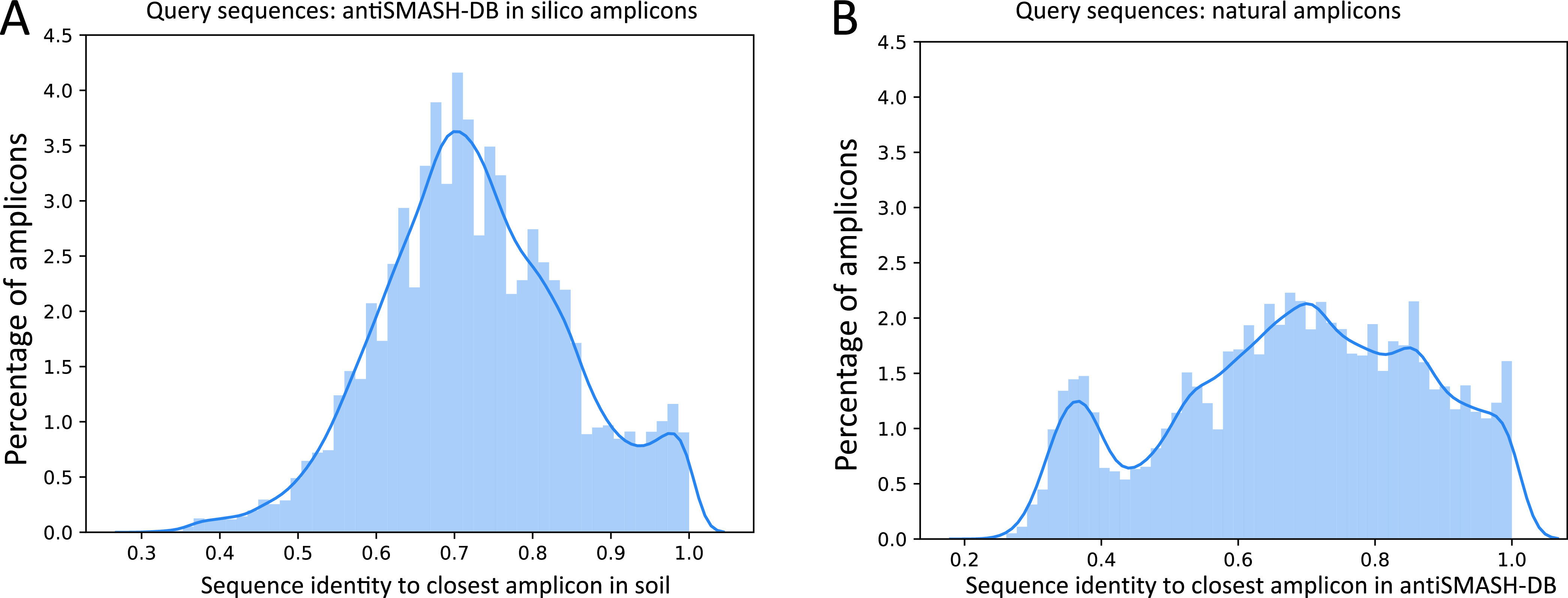
Sequence distance between nAMPs and antiSMASH-DB *in silico* amplicons. (A) Histogram showing the distribution of best matches (highest percentage identity at protein level) between each nAMP and the antiSMASH-DB *in silico* amplicon database. (B) Histogram showing the distribution of best matches (highest percentage identity at protein level) between each antiSMASH-DB *in silico* amplicon and the nAMPs.

Functional amplicon sequencing of A domains can achieve better coverage of domains from rare BGCs than metagenomics with the same sequencing volume. This is reflected by the high diversity of domains found in natural amplicons (nAMPs), with 40,005 unique amplicons at the protein level, compared to that with the shotgun assembly that yielded 8,762 unique *in silico* amplicons at the protein level. Remarkably, we observed that the number of unique sequences present in all our samples surpasses the diversity contained in antiSMASH-DB (24,085 AMPs), the largest available annotated database for natural product-encoding BGCs that contains sequence data for 32,548 BGCs from 24,776 microbial genomes. To highlight the importance of environmental sampling efforts, we further matched the nAMP sequences to *in silico* amplicons from antiSMASH-DB. We found that most sequences were matched at or above 70% identity. However, there were 162 instances of A domains with <30% amino acid sequence identity to their closest representative in the database. These domains, while still matching the Pfam domain, can potentially harbor novel functions, such as incorporation of different amino acids, or may simply belong to rare and uncharted BGCs. The percentage identity of nAMPs to the closest antiSMASH-DB AMP follows a normal distribution, with a peak to the right accounted for by (near-)perfect matches to previously sequenced clusters (Fig. 2A).

To evaluate the impact of the primer bias on the observed amplicon diversity, we performed an inverse analysis by identifying the closest match of *in silico* amplicons from antiSMASH-DB to the nAMPs from the soil, as the first is not affected by primer bias. The results revealed a bimodal distribution (Fig. 2B and Table S2). The leftmost mode includes amplicons not present in the samples as well as amplicons that might be present in the samples but absent in the nAMP set because of their poor match to the primer sequences. Still, the majority of the *in silico* amplicons from antiSMASH-DB had a match in our sample of >60% sequence identity. This indicated that the primer bias, despite being present, does not prevent the majority of the known sequence diversity of adenylation domains from being represented in the functional amplicon data. These results confirm the high value of functional amplicon sequencing studies in charting the biosynthetic potential of environmental niches. Based on these results we see that with limited primer bias we can still get substantial coverage of nAMPs.

10.1128/mSystems.01116-20.3 TABLE S2 Statistical tests for Fig. 2B. Download Table S2, XLSX file, 0.1 MB. Copyright © 2021 Tracanna et al. 2021 Tracanna et al. https://creativecommons.org/licenses/by/4.0/ This content is distributed under the terms of the Creative Commons Attribution 4.0 International license.

### The dom2BGC pipeline facilitates automated annotation and networking of functional amplicons.

**FIG 3:**
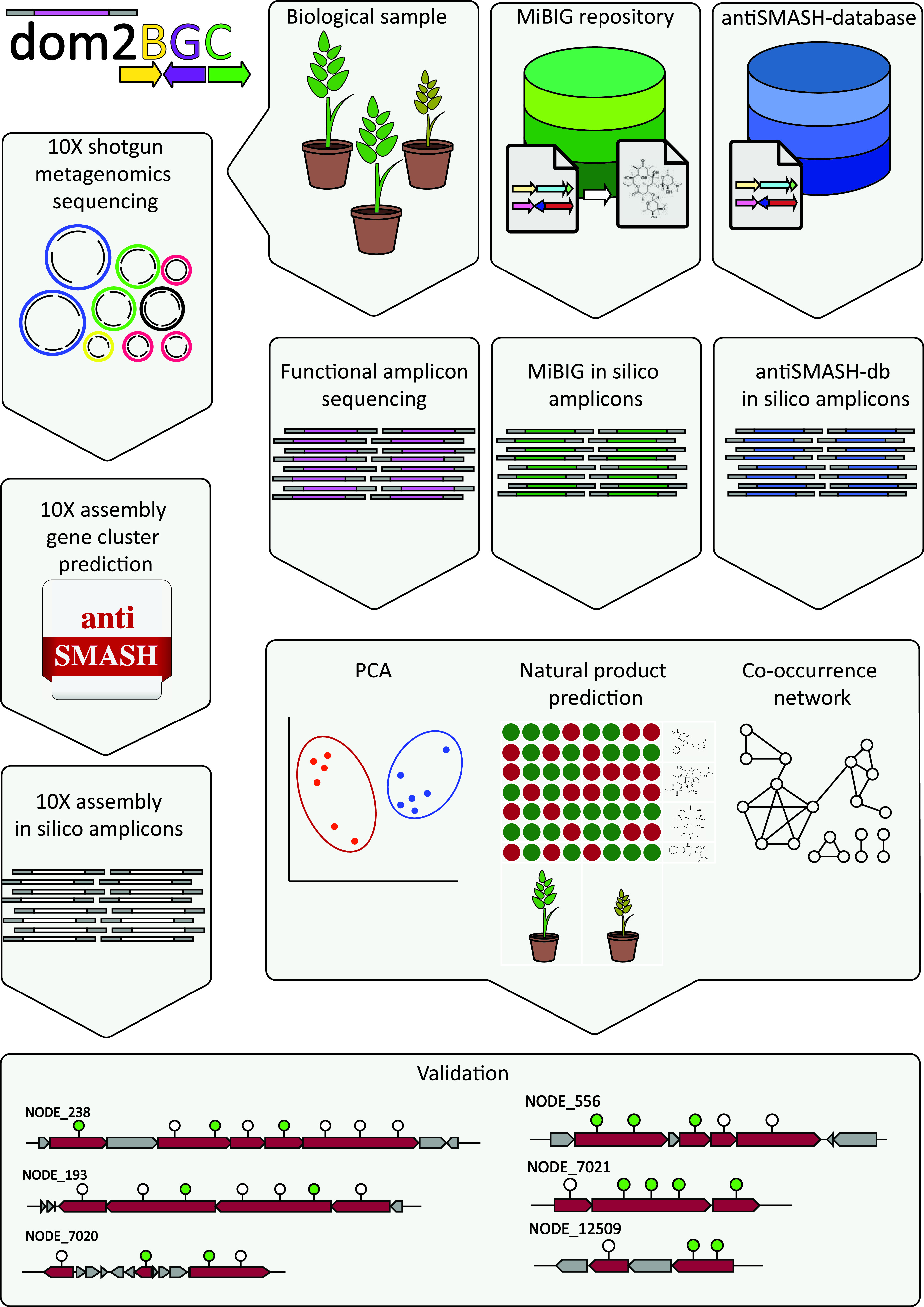
dom2BGC annotation pipeline and validation process. Amplified sequences from the rhizosphere are translated to nAMPs as per Materials and Methods and have been annotated through comparison with *in silico* amplicons from MIBiG and antiSMASH databases. Richness and community composition measures are used to assess their associations with phenotype and treatments. Co-occurrence patterns of amplicons which share similarity to the same reference BGCs were used to predict presence of (homologues of) known BGCs. Finally, in this study, a shotgun metagenomic assembly from one of the soil samples was used to confirm the presence of these predicted gene clusters from the amplicon data.

Current tools for the annotation of functional amplicons (eSNaPD [12] and NaPDoS [13]) have limited applications or rely on laborious processes which require expensive laboratory automation of bacterial artificial chromosome (BAC) clone library approaches (CONKAT-seq [14]). To harness the potential of A domain functional amplicons in soils, we developed dom2BGC, a pipeline to add taxonomical, functional, and product annotation to amplicon sequences and validate some of the predicted clusters using shotgun metagenomics assembly data. Within dom2BGC (Fig. 3), amplicons are matched to antiSMASH-DB and MIBiG, two natural product BGC databases, and annotations are transferred to the query amplicons when hits are reported above a user-set threshold (default, 95% identity). Diversity measurements and community structure relationships between samples are calculated and visualized in a series of automatically generated figures (examples, Fig. 2 and see Fig. 5 for an example network). Finally, a co-occurrence network of amplicons across the samples is created. Neighboring amplicons mapping onto domains of known clusters from antiSMASH-DB or MIBiG are considered domains which potentially belong to the same original cluster. This information can then be used in designing further experiments to validate the putative functions of the identified clusters.

To identify known natural product BGCs in the microbial communities, a total of 3,239 *in silico* amplicons were generated from MIBiG products entries (MIBiG amplicons [MAMPs]). Of these, 1,312 unique nAMPs, corresponding to 8% of the total, were matched and associated with a BGC for a known natural product. Notably, the most abundant known BGC annotated encodes the biosynthesis of pyoverdine; this NRPS gene cluster is widespread among Pseudomonas species, which are also common members of the rhizosphere. Still, even for MIBiG entries with a perfect match and consistent coverage across samples, not all of the A domains present in the reference cluster amplified. This illustrates how functional amplicon sequencing provides deep coverage of biosynthetic diversity across microbiome samples but also misses certain domains because of mismatches between oligoprimers and the target sequence or other PCR biases. This is partially balanced by the fact that most NRPS gene clusters encode multiple A domains, which increases the chance that at least one of these regions is amplified. As for database coverage, 119 of 860 entries with an adenylation domain in MIBiG had at least one amplicon from our data mapping to one of its domains with >90% amino acid identity across their lengths. This is testament to the extensive natural product potential of soil microbial communities.

To investigate the taxonomical and gene cluster class distributions of nAMPS, a total of 40,211 *in silico* amplicons were generated from antiSMASH-DB BGCs (aSAMPs) and used to annotate 5,531 nAMPs (corresponding to 29.9% of total reads), linking them to 1,443 different BGCs. This annotation rate constitutes about a 4-fold increase compared to the numbers of nAMPs that were annotated using MIBiG as reference.

### Disease suppression is not associated with increased adenylation domain diversity but shows a distinct community structure.

**FIG 4:**
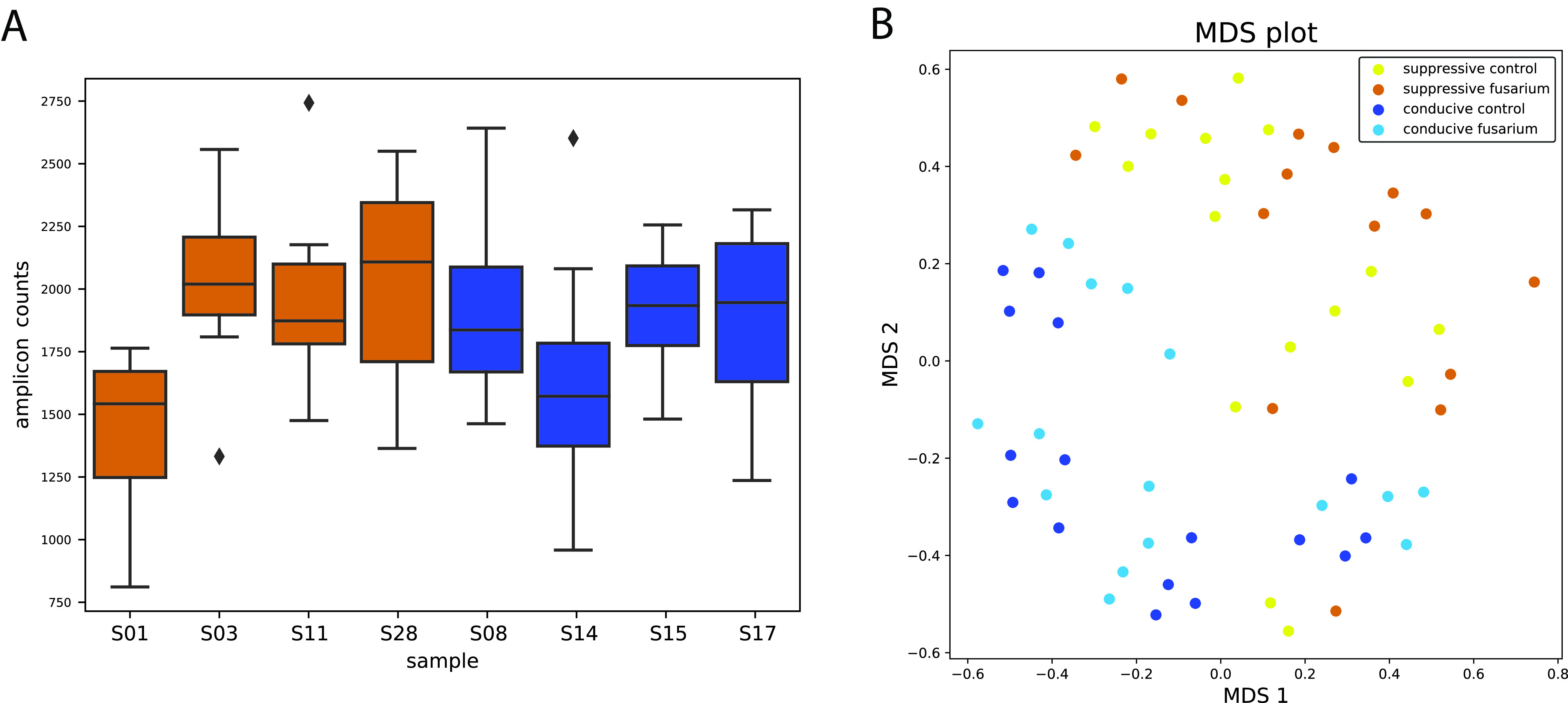
Community diversity and composition. (A) Adenylation domain richness across suppressive (orange bars) and conducive (blue bars) soils, calculated as unique sequences. (B) Visualization of the adenylation domain community composition with multidimensional scaling.

There is great need for diagnostic tools to assess the disease-suppressive potential of agricultural soils based on their microbial and functional compositions. In a recently published paper, Yuan et al. (15) explored in a meta-analysis the potential of 16S and internal transcribed spacer (ITS) amplicons as predictors of disease occurrence. Since A domain functional amplicon data showed more distinctive patterns than 16S data between soils with conducive and suppressive phenotypes (5), we set out to explore if it might be feasible to use functional amplicon sequencing as a diagnostic tool of disease suppressiveness. To test the possible association of within-sample amplicon diversity measures with the suppressive phenotype, we calculated within-sample richness, evenness, and phylogenetic diversity (PD) for all samples based on observed unique amplicons, Simpson *e*, and Faith PD, respectively. Wilcoxon rank sum tests showed no significant association of alpha diversity measures with the presence of the pathogen or with the suppressive phenotype for any of these metrics (Fig. 4A).

Several studies have associated overall microbial species richness or evenness in the soil and rhizosphere with disease suppressiveness (16–20). In other studies, however, this was not the case, and suppressiveness was associated with the abundance/enrichment of specific genera or functions (21, 22). Here, we note that suppressive soils were both among the most and least diverse in terms of NRPS A domains, which highlights the importance of availability of samples from multiple sources that share the same phenotype before drawing conclusions on the role of community diversity in disease suppression.

In a multidimensional scaling (MDS) analysis, suppressive soils did form a distinct group based on their community profile (Fig. 4B) with significant grouping, suggesting that similar community NRPS profiles can indeed be associated with the suppressive phenotype based on unweighted UniFrac (permutational multivariate analysis of variance [PERMANOVA] *P* value = 0.010, analysis of similarity [ANOSIM] *P* value = 0.010). This could indicate that the observed phenotype is caused by a single or limited number of pathways, not detectable with overall richness or abundance measurements, that directly interfere with a pathogen’s ability to colonize the rhizosphere and initiate root penetration and disease.

Thus, it appears that sequencing the A domain community composition has the potential to become a predictive tool for diagnosing soil suppressiveness. Nevertheless, we should emphasize that our study is based on only one host-pathogen system (wheat and Fusarium culmorum) and a collection of eight soils. Still, the fact that the production of compounds by NRPS and PKS enzymes plays crucial roles in other disease-suppressive soils (22–31) supports this proposition. This method has to be further developed and validated in the future through the inclusion of more host-pathogen systems and soils suppressive to other soilborne fungal pathogens.

### Suppressive soils are enriched in cyclic peptide-associated A domains.

Adenylation domains activate and incorporate specific amino acids in the growing nonribosomal peptide during synthesis by an NRPS assembly line. The substrate specificity for different A domains is determined by a restricted number of residues in their sequence (32). A domains incorporate a large variety of both proteogenic and nonproteogenic amino acids, which facilitate the structural diversity of the final peptide products. We reasoned that prediction of the substrate specificities of the domain amplicons detected in suppressive and conducive rhizosphere samples could provide new insights into the abundance and diversity of different products, and we trained a classifier to predict these specificities (see Materials and Methods). Intriguingly, we found predicted threonine-specific domains to be significantly more common in suppressive soils than in conducive soils (rank sum test *P* value < 0.001) (full result table in Table S3). This is particularly interesting as threonine is an amino acid commonly involved in lactone ring formation of cyclic and branched cyclic (lipo)peptides. Such peptides have a large variety of natural functions, which encompass, among others, the induction of systemic resistance in plants to fungal infection and direct antifungal activity (6, 33–39).

10.1128/mSystems.01116-20.4 TABLE S3 Cumulative relative abundance of predicted amino acid substrates in suppressive and conducive rhizosphere soils. Rank sum statistics were calculated using relative counts of appearances of amplicons annotated to the monomer for calculating ranks. Download Table S3, XLSX file, 0.1 MB. Copyright © 2021 Tracanna et al. 2021 Tracanna et al. https://creativecommons.org/licenses/by/4.0/ This content is distributed under the terms of the Creative Commons Attribution 4.0 International license.

### Reconstruction of 31 gene clusters from amplicon data using domain annotation and co-occurrence pattern analysis.

**FIG 5:**
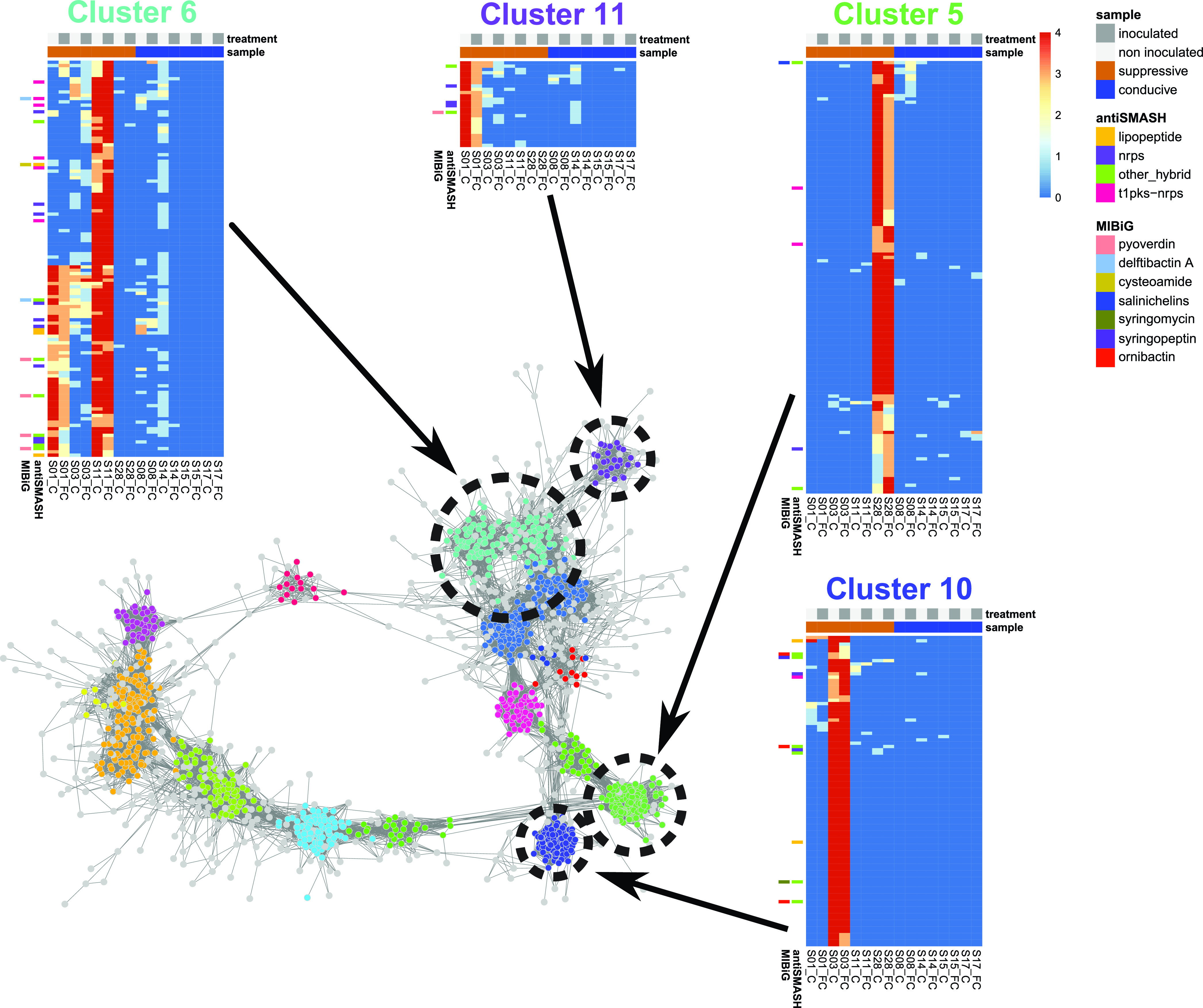
Domain co-occurrence network showing clusters associated with soil suppressiveness. For each of the four clusters (5, 6, 10, and 11), a heat map shows the distribution of A domains across the samples. The heat map color scale represents the numbers of replicates in which the A domain occurred (from dark blue [absent] to red [present in all four replicates]). Upper color bars in the heat maps describe samples: light gray, noninoculated; dark gray, inoculated with pathogen and disease suppressiveness; orange, suppressive; blue, conducive. The left side of each heat map shows which A domains were annotated using the MIBiG or antiSMASH databases with color bars. Color of the bars indicates a compound or compound class shown in the legend.

**FIG 6:**
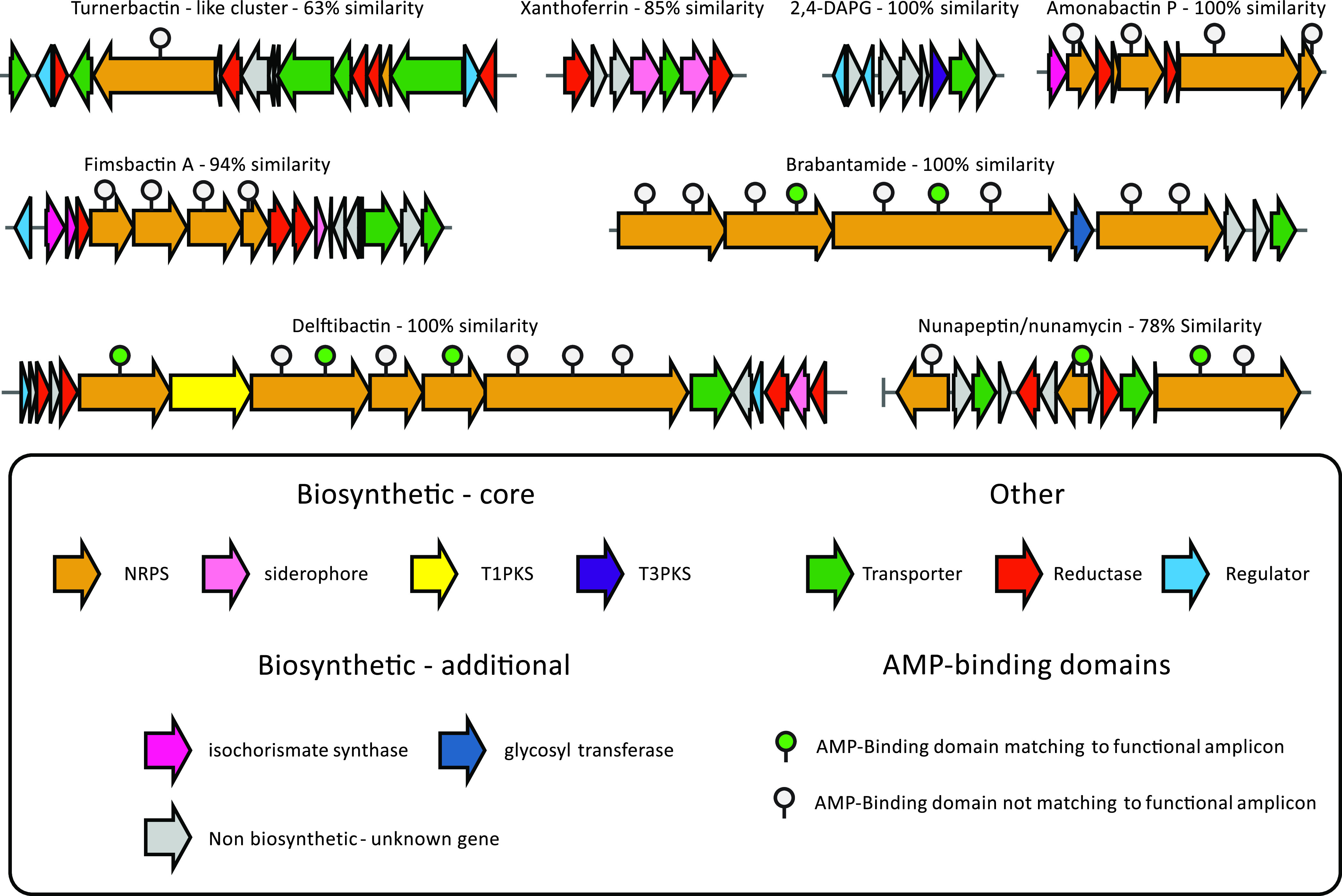
Selection of known BGCs predicted in the rhizosphere metagenome of suppressive soil S11. Arrows represent predicted genes and are color coded based on their annotated function. AMP-binding domains matching to functional amplicons are highlighted as described in the legend.

Co-occurrence of domains across the soil samples was used to build a pairwise co-occurrence matrix as described in Materials and Methods. A strict filter was applied to remove spurious correlations. To this end, we kept only the Spearman correlations above the 99th percentile, which resulted in a co-occurrence network containing 1,618 amplicons. Associations of co-occurring amplicons into putative BGCs were predicted only for co-occurring amplicons which share annotation to one or multiple references; this resulted in the reconstruction of 31 gene clusters (see Table S4). These clusters belonged to multiple taxonomical groups, namely, Pseudomonas, *Delftia*, *Streptomyces*, *Variovorax*, *Burkholderia*, and *Collimonas*. To validate putative network clusters, we generated 8,762 *in silico* amplicons from our 10× shotgun metagenome assembly as described above. Two of the 31 reconstructed gene clusters matched to known gene cluster products predicted from the metagenome: the BGCs for nunamycin and delftibactin from Pseudomonas and *Delftia,* respectively, as shown in Fig. 5 and 6.

10.1128/mSystems.01116-20.5 TABLE S4 Annotated MIBiG clusters with counts in all suppressive and all conducive soils. Download Table S4, XLSX file, 0.1 MB. Copyright © 2021 Tracanna et al. 2021 Tracanna et al. https://creativecommons.org/licenses/by/4.0/ This content is distributed under the terms of the Creative Commons Attribution 4.0 International license.

### Overview of the BGCs associated with suppressive soils.

Next, we identified in more detail the BGCs detected in the wheat rhizosphere microbiome from suppressive soil S11. To this end, we used antiSMASH to identify BGCs in the 10× shotgun metagenome assembly of this soil. This resulted in 991 predicted BGCs from multiple gene cluster families (GCFs) associated with various known compounds. Notable compounds include siderophores such as turnerbactin, delftibactin, fimsbactin, xanthoferrin, and amonabactin, lipopeptides such as nunamycin/nunapeptin and brabantamide (27, 40–45), and known antifungal compounds such as 2,4-diacetylphloroglucinol (26). This array of candidate clusters offered an initial insight into putative mechanisms associated with the disease-suppressive phenotype, in which one or multiple compounds may inhibit simultaneously or sequentially the growth of the invading pathogen and suppress root infection.

### Analysis of siderophores and lipopeptides associated with observed phenotypes.

As expected, our MIBiG-based annotations show that a considerable portion of the amplicons (955 of 5,531) mapped to Pseudomonas A domains. A domains from this study mapped to BGCs belonging to 68 different genera and 208 bacterial species (Table S2). With these taxonomic annotations obtained from dom2BGC, it was possible to identify taxonomic patterns of adenylation domains associated with soil disease suppressiveness. Multiple species known for their biosynthetic potential and for involvement in disease suppressiveness in other systems were significantly enriched in suppressive soils at high taxonomical resolution (Table S4). This suggests that these bacteria, which were previously found to exhibit antifungal activity, might also play a role in the disease suppressiveness against *F. culmorum* in wheat.

DBscan clustering of the A domain co-occurrence network produced 16 clusters. Among these clusters, 4 were associated with at least one suppressive soil. The most interesting subnetwork (Fig. 5, cluster 6) has amplicons associated with suppressive soil S11 and partially with soil S01, with some amplicons present across three suppressive soils. Three separate domain clusters were reconstructed within this subnetwork, with all three matching BGCs encoding the production of known siderophores, namely, pyoverdine from Pseudomonas, scabichelin from *Streptomyces*, and delftibactin from *Delftia*. All of these were associated with suppressive soil S11, and the last one was associated with suppressive soil S01 as well. Siderophores are a group of secondary metabolites produced by microorganisms in iron-limited environments such as soil. These metabolites form complexes with insoluble iron, facilitating the uptake of this iron by microorganisms. Often, competition for iron is a central process in soil systems with neutral to high pH (46–50). Siderophores and competition for iron were found to be involved in soil disease suppression mechanisms against Fusarium wilt (23–25, 51, 52), take-all disease in wheat (53, 54), and damping-off sugar beet (22).

The concentrations of soluble iron in eight tested soils, as assayed in our previous study (4), ranged from 0.01 mg/kg in soil S17 to 0.11 mg/kg in soil S11 with the exception of soil S03, where the concentration was much higher and reached 0.45 mg/kg. The high iron concentration in soil S03 can be explained by its low pH (5.28), which increases the solubility of oxidized iron. All other soils had a neutral pH (7.13 to 7.82) or were only slightly acidic (soils S01 and S08, pH 6.22 and 6.87, respectively) (Table S5 and reference 4). We observed that the broad presence of siderophores was not limited to environments with a low availability of iron. Those results do not indicate a simple connection between the concentration of soluble iron and soil disease suppressiveness against *F. culmorum*. Nevertheless, the production of siderophores is so widespread among microorganisms in soil systems that we can consider it a primary process in ecosystem functioning consequently indispensable for soil disease suppressiveness.

10.1128/mSystems.01116-20.6 TABLE S5 Chemical properties of tested soils. Download Table S5, XLSX file, 0.1 MB. Copyright © 2021 Tracanna et al. 2021 Tracanna et al. https://creativecommons.org/licenses/by/4.0/ This content is distributed under the terms of the Creative Commons Attribution 4.0 International license.

The network hub associated with suppressive soil S03 (Fig. 5, cluster 10) contains three predicted reconstructed gene clusters taxonomically assigned to *Burkholderia*, *Collimonas*, and Pseudomonas. The *Burkholderia* and *Collimonas* clusters matched to multimodular NRPSs with no known associated natural product, while the reconstructed cluster from Pseudomonas matched to the syringafactin BGC. Finally, the pyoverdine BGC from Pseudomonas was recovered from a smaller amplicon subnetwork (Fig. 5, cluster 11). While the consistent recovery of the pyoverdine BGC in multiple hubs is expected given its ubiquity in rhizosphere-associated pseudomonads, the recovery of the delftibactin and scabichelin BGCs and their association to two suppressive soils emphasize the contribution of different kinds of siderophores to disease suppression. Our results were further confirmed by the prediction of a delftibactin BGC in the associated shotgun metagenome assembly from soil S11 with antiSMASH, which has an almost perfect match with the delftibactin BGC in MIBiG (Fig. 6). The largest suppressive sample-associated subnetwork by number of amplicons (Fig. 5, cluster 5) possesses an individual cluster matching the scabichelin BGC from Streptomyces scabies. This siderophore has been found to be produced by previously reported Fusarium-suppressive strains (55). The reconstruction of separate instances of the same BGC suggest that the underlying amplicons belong to variants of the scabichelin cluster present in different rhizosphere communities.

All in all, the results suggest an association of siderophore BGCs with the disease-suppressive phenotype across the soils studied. They also point to a possible functional redundancy that should be validated in future work: in some soils, a suppressive function might be mediated through the production of some siderophores (e.g., delftibactin), while in other soils, the same function might be mediated by other natural products (e.g., scabichelin).

Based on the MIBiG database, 15 lipopeptides were annotated in our samples. Figure S2 presents the distribution of these compounds among suppressive and conducive soils. Interestingly, most annotated lipopeptides are much more abundant in conducive soils, especially in soil S17. Many of these lipopeptides are connected to bacterial plant pathogens and act like pathogenicity factors (for example, syringafactin, tolaasin, and sessilin), while others have been implicated in soil disease suppressiveness and antagonistic interactions with fungi (for example, nunamycin and thanamycin) or inhibiting the formation of bacterial biofilms (for example, white-line-inducing principle [WLIP], entolysin, putisolvin, and xantholysin A). Many of the A domains that are part of NRPS BGCs of plant-pathogenic bacteria are also part of NRPS BGCs of nonpathogenic bacteria (56). Isolation of the bacteria harboring these BGCs and subsequent genetic, genomic, transcriptomic, and mutational analyses will be needed to determine the identity as well as any functional significance of these BGCs in suppressiveness.

10.1128/mSystems.01116-20.8 FIG S2 Phylogenetic tree of the natural amplicons analyzed in this study. On the outer ring, colors represent predicted amino acid specificity and amino acid group specificity as detailed in Text S1. Download FIG S2, PDF file, 1.6 MB. Copyright © 2021 Tracanna et al. 2021 Tracanna et al. https://creativecommons.org/licenses/by/4.0/ This content is distributed under the terms of the Creative Commons Attribution 4.0 International license.

10.1128/mSystems.01116-20.1 TEXT S1 Description of methods related to wheat growth conditions and inoculation, disease suppressiveness assays, PCR amplification and sequencing, rhizosphere DNA extraction and 10× metagenomic assembly, diversity measures used in the dom2BGC pipeline, and feature extraction for substrate specificity prediction. Download Text S1, PDF file, 0.2 MB. Copyright © 2021 Tracanna et al. 2021 Tracanna et al. https://creativecommons.org/licenses/by/4.0/ This content is distributed under the terms of the Creative Commons Attribution 4.0 International license.

### Conclusions.

Our study provides novel insights into the NRPS AMP-binding domain diversity of agricultural rhizosphere samples. Remarkably, the diversity of the set of unique amplicons from this rhizosphere collection equals the level of diversity of adenylation domains found across all publicly available genomes. Annotation rates for nAMPs were generally low, which highlights the incredible potential of plant-associated microbiomes for discovering novel natural products. We report significant community structure overlap among suppressive rhizobacterial adenylation domain profiles, and we generated new hypotheses regarding possible roles for siderophores in disease suppression against Fusarium culmorum. We also developed a pipeline for taxonomic and functional annotation of NRPS amplicons without the requirement of a BAC clone library. The dom2BGC pipeline can be extended to and currently supports annotation of any natural product-associated domain that occurs multiple times within a BGC and, to some extent, for any BGC-associated domain. We validated the amplicon clustering results by reconstructing the delftibactin BGC, a siderophore associated with suppressive soils, using a combination of amplicon sequencing and novel 10× genomics shotgun metagenomics sequencing. We conclude that combining functional amplicon sequencing and shotgun metagenomics represents a powerful approach to probe complex microbiome-associated plant phenotypes and to generate new hypotheses on the functional roles of microbial metabolites in microbe-microbe and microbe-host interactions.

## MATERIALS AND METHODS

### Soil collection.

Eight soil samples (S01, S03, S08, S11, S14, S15, S17, and S28) were collected from 3-m squares located at the center of each agricultural field in January to April 2017. In this area, topsoil cores of approximately 30 cm in depth were collected. Soils were air dried at room temperature, homogenized, sieved through a 4-mm mesh sieve, and stored at 4°C. Soil S28 was additionally flaked after drying using a jaw crusher (type BB-1; Retsch, Germany). Detailed descriptions of the soil samples are included in our previous study (4).

### Disease suppressiveness assay and A domain amplification.

Wheat growth conditions, pathogen inoculation, the suppressiveness assay, A domain amplification and sequencing are described in detail in Text S1 in the supplemental material. Briefly, wheat seedlings were transferred to substrate containing one of the eight tested soils and challenged with pathogenic *F. culmorum* PV using untreated plants as a control; each combination had 12 replicates. After 3 weeks, wheat plants were inspected for disease symptoms and given a disease index describing the severity of infection from 0 (healthy plant) to 5 (heavily diseased), as in our previous work (4). Rhizosphere DNA was isolated from 4 randomly chosen replicates per treatment. NRPS adenylation domains were amplified using A3F and A7R primers (57) using Q5 polymerase.

### A domain amplicon preparation.

Barcoding and sequencing of the A domain amplicons were performed at BaseClear (Leiden, The Netherlands) using Illumina MiSeq, which generated 4,181,437 paired-end reads of 250 bp in length. Sequences were demultiplexed and adapters trimmed using Qiime2 (58). Quality filtering and denoising were performed with DADA2 (59). Nucleotide sequences were translated to amino acid sequences (for all reading frames) with transeq from the EMBOSS suite (60). Forward sequences were aligned with the AMP-binding domain hidden Markov model (HMM) profile PF00501 from the Pfam database (version 27) (61) using hmmsearch from the HMMer package [version 3.1] (62). The output table was parsed to retain only the conserved amino acids in the sequence corresponding to “match” states with the HMM profile. Protein sequences shorter than 66 amino acids were discarded. The resulting prealigned amplicon sequences from the natural source are referred to as nAMPs (natural amplicons) to distinguish them from the *in silico* amplicons used for their annotation.

### 10× metagenome sequencing.

DNA extraction, sequencing, and assembly are described in detail in Text S1. Briefly, 10× Genomics Chromium was used to generate a read cloud library from high-quality rhizosphere DNA and subsequently sequenced on an Illumina NovaSeq 6000.

### Feature extraction from amplicons for substrate specificity prediction.

In all, 1,029 experimentally validated bacterial NRPS A domains from the MIBiG database were used as a training set. Training set sequences were aligned to the AMP-binding (PF00501) HMM, and the range of 34 residues that aligned with positions 210 to 243 (PheA) were extracted. All duplicates and any sequences for which there were fewer than seven training examples for a given amino acid substrate were removed from the data set, leaving a training set of 848 sequences (available at https://git.wur.nl/traca001/dom2bgc). Each of the 34 residues was encoded as a vector of 15 physicochemical properties, including hydrophobicity, secondary structure, size, and polarity (5). The full vector of 510 features was used to train separate random forest models to predict amino acid monomer specificity and broad substrate groups using the SKLearn package (version 0.20.2) (6) in Python [version 3.7.3].

Each random forest classifier was randomly split with class-specific stratification into 90% training and 10% test. Model parameters were tuned based on an out of bag (OOB) score for the training set over 3 iterations. Overfitting was limited by pruning the tree depth to a maximum of 20. The number of features randomly sampled as candidates for each split was set to the default (square root of the number of predictors). The random forest was grown to a size of 1,000 trees.

Final models for monomer and broad substrate group classification were used to make predictions for the 51,914 soil amplicon sequences. Sequences with a prediction probability score of less than 0.5 were labeled as “no confident prediction.” Approximately 65% of the broad substrate groups and 49% of the monomers were predicted with confidence.

### dom2BGC pipeline.

**(i) Generation of *in silico* amplicons.** To generate a reference data set of NRPS functional amplicons, A domain sequences were extracted from antiSMASH-DB and MIBiG BGCs. In dom2BGC, *in silico* amplicons are created by searching these sequences using hmmsearch with the A domain hidden Markov model (HMM) profile from Pfam (PF00501) (61). This produces reference sequences aligned to the HMM profile. To produce *in silico* amplicons comparable to the nAMPs, the alignment matching the nAMP match coordinates is extracted. This process creates *in silico* amplicons that are prealigned to the nAMPs, which allows for quick matching between nAMPs and *in silico* AMPs using pairwise identity. Annotations available for *in silico* amplicons are stored to be transferred to any nAMPs matching with it. Currently supported annotations include, where available, the taxonomy of the source organism, the BGC type annotation based on antiSMASH predictions, and the name of the natural product for which the production is encoded in the BGC (for domains extracted from MIBiG entries [63]). Calculations for diversity measures and community composition are described in Text S1.

**(ii) Amplicon matching and annotation.** Each nAMP is matched to an *in-silico* amplicon if it shares 90% or more of its amino acid sequence with the reference over the full amplicon length. For nAMPs matching to multiple *in silico* AMPs within a reference database, all entries are recorded. In case of multiple nAMPs matching an individual *in silico* amplicon, all matched nAMPs are grouped for evaluation of presence-absence patterns and abundance of the *in silico* amplicon.

In dom2BGC, amplicons are taxonomically annotated at the lowest rank available. In case of annotation to a reference BGC with a different taxonomic annotation, dom2BGC assigns the amplicon to the lowest common ancestor of the matching references. In addition, information from the reference cluster on the gene cluster family is passed on to the matching amplicon. This annotation is based on antiSMASH classification rules for predicted gene clusters. Possible annotations include NRPS, lipopeptides, hybrid PKS, and more. In case of an amplicon matching with reference clusters belonging to different gene cluster families, dom2BGC reports all matches.

**(iii) Co-occurrence network creation and analysis.** Pairwise co-occurrence patterns of nAMPs are calculated using Spearman rank correlation of presence-absence patterns using numpy meshgrid. To filter out spurious relationships, the correlation network contains only the strongest correlations in the 99th percentile among abundant nAMPs. In the resulting network, amplicons are nodes and edges are drawn based on co-occurrence. Clustering within the network to define BGC hubs is performed with DBscan. These BGC hubs, comprising highly correlated nAMPs, are inspected for nAMP annotation enrichment. Cluster nodes and first-degree neighbors annotated to the same reference gene cluster are further selected as putative gene clusters. Networks are visualized in Cytoscape (64), and putative clusters are reported in a separate tab-separated file.

### Data availability.

Raw sequence data that support the findings of this study have been deposited in NCBI under project number PRJNA719981.
