# Supplementary figures and images for: Dissecting Disease-Suppressive Rhizosphere Microbiomes by Functional Amplicon Sequencing and 10× Metagenomics
Source: mSystems. 2021 Jun 8;6(3):e01116-20. doi: 10.1128/mSystems.01116-20 (PMC8269251; doi:10.1128/mSystems.01116-20)

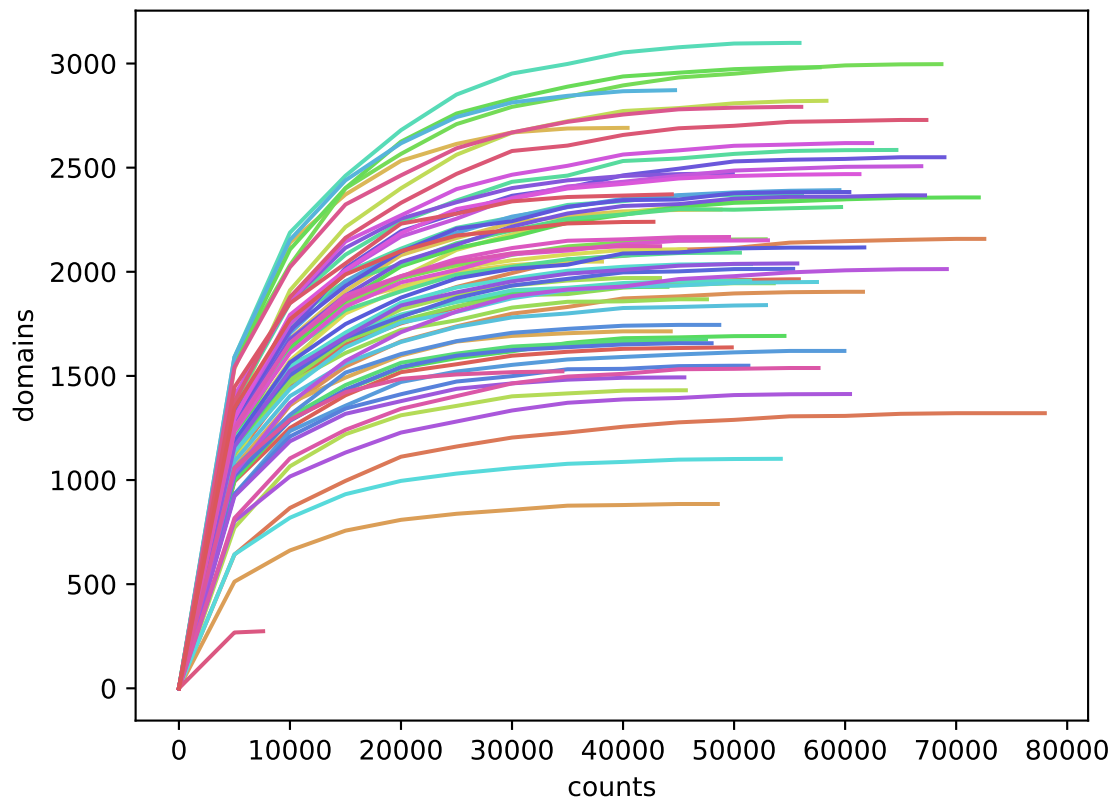

Supplement: FIG S1 [file msystems.0116-20-sf001.pdf]

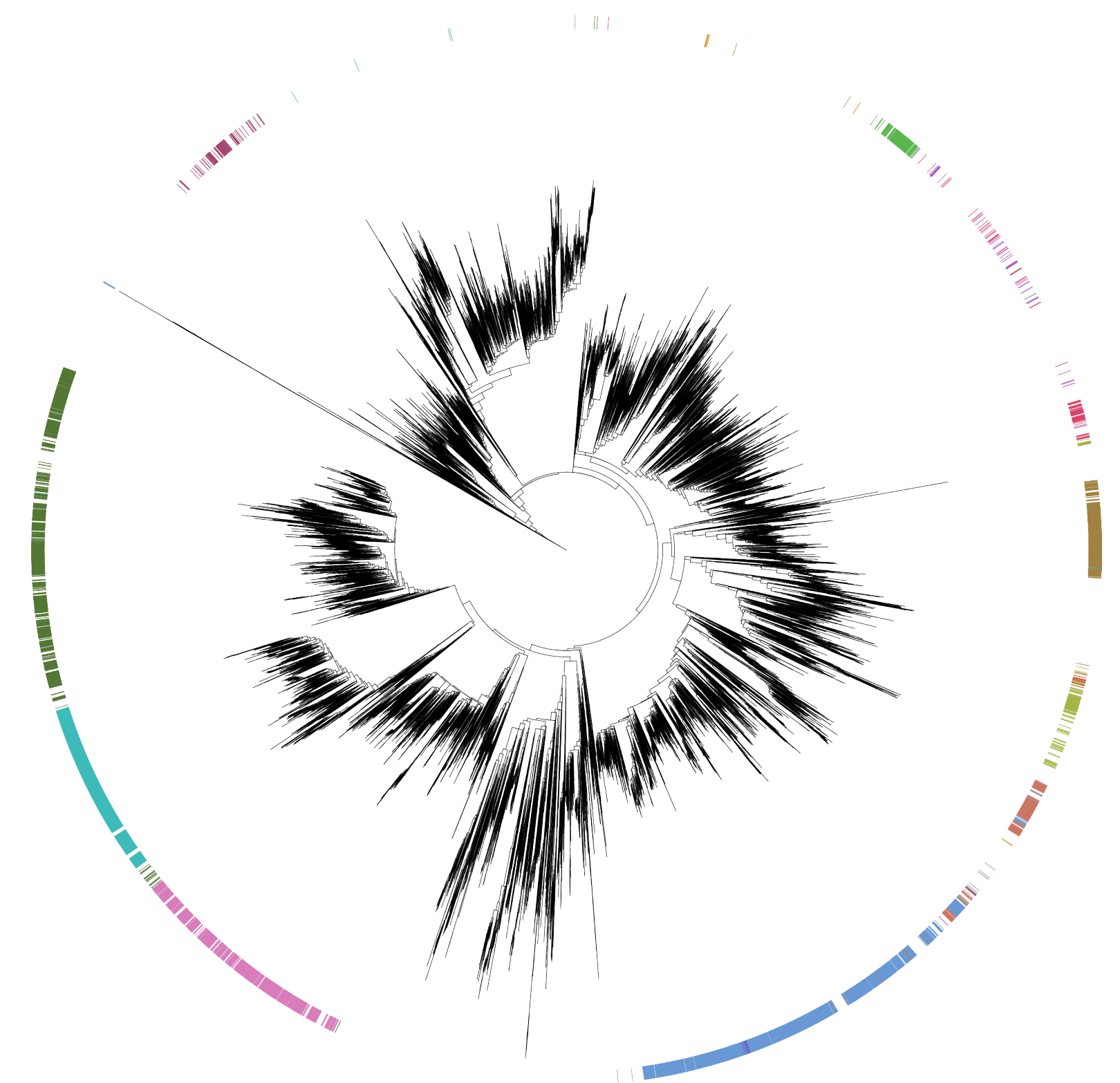

|     |     |     |     |     |
|-----|-----|-----|-----|-----|
| Thr | Phe | Dab | Val | Leu |
| Tyr | Asn | Ser | Ala | Cys |
| Pro | Glu | Asp | Gly | Gln |

Supplement: FIG S2 [file msystems.0116-20-sf002.pdf]
