# Supplementary material for: Dissecting Disease-Suppressive Rhizosphere Microbiomes by Functional Amplicon Sequencing and 10× Metagenomics
Source: mSystems. 2021 Jun 8;6(3):e01116-20. doi: 10.1128/mSystems.01116-20 (PMC8269251; doi:10.1128/mSystems.01116-20)
Supplement: TEXT S1 [file msystems.0116-20-s0001.pdf]

Supplementary Materials & Methods for:

Dissecting disease-suppressive rhizosphere microbiomes by functional amplicon sequencing and 10X metagenomics

Authors:
Vittorio Tracanna[1*], Adam Ossowicki[2*], Marloes L. C. Petrus[3], Sam Overduin[1], Barbara R. Terlouw[1], George Lund[4], Serina L. Robinson[5], Sven Warris[6], Elio G. W. M. Schijlen[6], Gilles P. van Wezel[2,3], Jos M. Raaijmakers[2,3], Paolina Garbeva[2#], Marnix H. Medema[1#]

1. Bioinformatics Group, Wageningen University and Research, Wageningen, The Netherlands
2. Microbial Ecology, Netherlands Institute of Ecology (NIOO-KNAW), Wageningen, The Netherlands
3. Microbial Biotechnology, Leiden Institute of Biology, Leiden, The Netherlands
4. Biointeractions and Crop Protection, Rothamsted Research, Harpenden, United Kingdom
5. BioTechnology Institute, University of Minnesota-Twin Cities, MN, USA
6. Bioscience, Wageningen University and Research, Wageningen, The Netherlands

*: contributed equally to this work
#: corresponding authors; email: p.garbeva@nioo.knaw.nl, marnix.medema@wur.nl

Wheat growth conditions and inoculation

Greenhouse experiments were performed in growth cabinets (MC 1750 VHO-EVD, Snijders Labs) at 20°C day and night, photoperiod 12 h day /12 h night and 60 % relative humidity. In these experiments, surface-sterilized and pre-germinated wheat seeds (JB Asano from Agrifirm, Netherlands) were used. After seedling emergence, plants were watered every second day and supplemented weekly with 0.5 Hoagland solution (1 ml per 80 cc of the soil, 0.5 M $Ca(NO_3)_2{\cdot}4H_2O$, 1 M KNO_3, 1 M KH_2PO_4, 0.5 M $MgSO_4{\cdot}7H_2O$ and 98.6 mM ferric EDTA).

As a reference, we used a dune soil collected near Bergharen, the Netherlands (BS) [26]. Before use, the Bergharen dune soil (BS) was air-dried, sieved and gamma-sterilized (Synergy Health Ede B.V., the Netherlands). Sub-samples from the eight agricultural soils were gamma-sterilized under the same conditions for later use. Prior to the experiment, all soils were microbially "activated" by growing wheat seedlings for two weeks followed by removal of the root material and thoroughly mixing the remaining soil.

The soil-borne pathogen *F. culmorum* PV was grown on ½-strength PDA medium (Oxoid, the Netherlands). For plant inoculation, the fungus was transferred to ¼-strength PDA and grown for 2 weeks at 20°C. After incubation, 6-mm-diameter agar plugs were cut from the border zone of the *Fusarium* hyphal culture and mixed with the growth substrate (1 plug per 10 cc). Controls were mock-inoculated with sterile ¼-strength PDA plugs without the pathogen.

Disease suppressiveness assay

In order to confirm the disease suppressive and conducive phenotypes observed in our previous study, we mixed the agricultural soils in a 3:7 (v/v) ratio with a mix of sterile BS and vermiculite (Agra-vermiculite, The Netherlands). Plants were grown in 380 cc soil per pot (7x7x8 cm), each containing three plants. Sterile BS with vermiculite mixture was used as a control. Soil samples and control were tested with and without addition of the pathogen inoculum. For each treatment, twelve replicates were used. After three weeks of plant growth, the wheat rhizosphere was collected and disease symptoms were assessed according to the methods described in our previous study (1). Briefly, for sampling the rhizosphere, plants were carefully removed from their pots. Soil loosely adhering to the roots was removed by gentle shaking. Four replicates in each treatment were used to isolate rhizosphere DNA. The roots and surrounding soil were placed on a sterile filter in a laminar flow cabinet and the soil particles adhering to the roots were collected using a brush. DNA was isolated with the DNeasy PowerSoil Kit (QIAGEN, the Netherlands) according to the manufacturer's protocol, using 0.25 g of rhizosphere soil. Samples were purified afterwards with the DNeasy PowerClean cleanup kit (QIAGEN, the Netherlands). The quality of DNA was assessed with Nanodrop (Thermo Scientific Nanodrop Products Inc., USA) and by gel electrophoresis.

PCR and sequencing

NRPS adenylation domains were amplified from 64 DNA samples with A3F-5'-GCSTACSYSATSTACACSTCSGG-3' and A7R-5'SASGTCVCCSGTSCGGTAS-3' primers (2) containing adapter sequences for barcoding. Amplification was performed with 200 ng of rhizosphere DNA per reaction using Q5 High-Fidelity DNA Polymerase (New England Biolabs, USA). The following PCR programme was used: 5 min 98 °C, 10 s 98 °C, 30 s 63.5 °C, 45 s 72 °C (35 cycles) and 5 min 72 °C. Afterwards, PCR products were purified using DNA Clean & Concentrator-5 kit (Zymo Research, USA).

Rhizosphere DNA extraction and 10X metagenome assembly

The rhizosphere of wheat grown in the highly suppressive soil sample S11 (Fig.1) was isolated by first removing soil loosely adhering to the roots and collecting the rhizosphere soil using a brush. The DNA

was isolated using a DNeasy PowerSoil Kit (QIAGEN, the Netherlands) according to manufactures protocol using 0.25 g of rhizosphere soil with small modifications. To avoid extensive fragmentation of DNA, instead bead beating, the samples were rotated gently for 30 min. DNA fractions from four technical replicates were pooled together. Pooled DNA sample was additionally purified using DNeasy PowerClean cleanup kit (QIAGEN, the Netherlands). Integrity of DNA was tested using Fragment Analyser (Agilent, USA) with the HS Extended Large Fragment method, showing fragment lengths of on average around 35000 bp. From the purified DNA, a read-cloud library was directly prepared on a 10X Genomics Chromium (Applied Bioinformatics WUR, Wageningen, NL) using 2,503,836 barcodes. This library was sequenced on an Illumina NovaSeq 6000 machine (Applied Bioinformatics WUR, Wageningen, NL) in a S2 flowcell, generating 70.04 Gbp of sequence data. Then, sequences were demultiplexed, subjected to quality control and barcoded with 10X Long Ranger v2.2.2. Additionally, barcodes detected within reads were trimmed using a custom script. This procedure was repeated using 2,434,601 barcodes, producing an additional 79.89 Gbp of sequence data. Both libraries were concatenated and before assembly digitally normalized using bbnorm [version 38.67] with mindepth and maxdepth set to 2 and 150 respectively due to memory constraints. AntiSMASH [v5.0] was run with default settings to predict BGCs across all contigs larger than 5kb.

dom2BGC pipeline - calculation of diversity measures

To compare different sample phenotypes to their community compositions, dom2BGC calculates different diversity measures and automatically generates figures for their interpretation. Standard measures include unique amplicons per sample (after clustering at protein level), as well as Simpson's Evenness (Simpson's E), which measures how dominant the more abundant amplicons are in a sample. Additionally, when a newick-formatted phylogenetic tree of the sequences is provided, dom2BGC calculates the Faith-PD diversity for all the samples. For community composition measures, unweighted UniFrac is chosen if a phylogenetic tree is provided and Bray-Curtis when no phylogenetic tree is available. Visualization is performed with boxplots for Simpson's E, unique amplicons and faith-PD, while multidimensional scaling and 3D PCA are used for unweighted UniFrac and Bray-Curtis.

Feature extraction from amplicons for substrate specificity prediction

51,914 A-domain amplicon sequences were aligned to the AMP-binding (PF00501) Hidden Markov Model (HMM). The alignment was visually inspected to ensure proper alignment with the HMM. To predict A-domain substrate specificity, the existing program NRPSPredictor2 (3) extracts 34 residues corresponding to residues within 8 Å of the PheA gramicidin synthetase active site (PDB ID: 1AMU) and uses them as features for classification (4). In the case of the amplicons, only part of the active site is amplified, specifically the region corresponding to positions 193 and 259 of the PheA sequence. Since the amplified region only covers the first 12 out of 34 active site residues, we investigated the use of a larger set of features to improve prediction accuracy. We tested model performance using the full range of residues between positions 210 – 243 (PheA) as features. Phylogenetic analysis of the resulting sequences indicated that there was not a strong taxonomic bias, i.e. the extracted residues still appeared to cluster based on amino acid specificity rather than source organism or gene cluster product.

References

1. Ossowicki A, Tracanna V, Petrus MLC, van Wezel G, Raaijmakers JM, Medema MH, Garbeva P. 2020. Microbial and volatile profiling of soils suppressive to *Fusarium culmorum* of wheat. Proceedings of the Royal Society B: Biological Sciences 287:20192527.

2. Ayuso-Sacido A, Genilloud O. 2005. New PCR primers for the screening of NRPS and PKS-I systems in actinomycetes: detection and distribution of these biosynthetic gene sequences in major taxonomic groups. Microb Ecol 49:10–24.

3. Röttig M, Medema MH, Blin K, Weber T, Rausch C, Kohlbacher O. 2011. NRPSpredictor2—a web server for predicting NRPS adenylation domain specificity. Nucleic Acids Res 39:W362–W367.

4. Conti E, Stachelhaus T, Marahiel MA, Brick P. 1997. Structural basis for the activation of phenylalanine in the non-ribosomal biosynthesis of gramicidin S. EMBO J 16:4174–4183.

5. Rausch C, Weber T, Kohlbacher O, Wohlleben W, Huson DH. 2005. Specificity prediction of adenylation domains in nonribosomal peptide synthetases (NRPS) using transductive support vector machines (TSVMs). Nucleic Acids Res 33:5799–5808.

6. Pedregosa F, Varoquaux G, Gramfort A, Michel V, Thirion B, Grisel O, Blondel M, Prettenhofer P, Weiss R, Dubourg V, Vanderplas J, Passos A, Cournapeau D, Brucher M, Perrot M, Duchesnay E. 2011. Scikit-learn: Machine Learning in Python. J Mach Learn Res 12: 2825-2830.
